# Estradiol differentially affects auditory recognition and learning according to photoperiodic state in the adult male songbird, European starling (*Sturnus vulgaris*)

**DOI:** 10.7717/peerj.150

**Published:** 2013-09-12

**Authors:** Rebecca M. Calisi, Daniel P. Knudsen, Jesse S. Krause, John C. Wingfield, Timothy Q. Gentner

**Affiliations:** 1 Department of Neurobiology, Physiology, and Behavior, The University of California, Davis, USA; 2 Department of Psychology, The University of California, San Diego, USA; 3 Neurosciences Graduate Program, The University of California, San Diego, USA

**Keywords:** Auditory learning, Auditory recognition, Songbirds, Photoperiod, Seasonality, Estradiol, European starling, *Sturnus vulgaris*, Learning, Fadrozole

## Abstract

Changes in hormones can affect many types of learning in vertebrates. Adults experience fluctuations in a multitude of hormones over a temporal scale, from local, rapid action to more long-term, seasonal changes. Endocrine changes during development can affect behavioral outcomes in adulthood, but how learning is affected in adults by hormone fluctuations experienced during adulthood is less understood. Previous reports have implicated the sex steroid hormone estradiol (E2) in both male and female vertebrate cognitive functioning. Here, we examined the effects of E2 on auditory recognition and learning in male European starlings (*Sturnus vulgaris*). European starlings are photoperiodic, seasonally breeding songbirds that undergo different periods of reproductive activity according to annual changes in day length. We simulated these reproductive periods, specifically 1. photosensitivity, 2. photostimulation, and 3. photorefractoriness in captive birds by altering day length. During each period, we manipulated circulating E2 and examined multiple measures of learning. To manipulate circulating E2, we used subcutaneous implants containing either 17-β E2 and/or fadrozole (FAD), a highly specific aromatase inhibitor that suppresses E2 production in the body and the brain, and measured the latency for birds to learn and respond to short, male conspecific song segments (motifs). We report that photostimulated birds given E2 had higher response rates and responded with better accuracy than those given saline controls or FAD. Conversely, photosensitive, animals treated with E2 responded with less accuracy than those given FAD. These results demonstrate how circulating E2 and photoperiod can interact to shape auditory recognition and learning in adults, driving it in opposite directions in different states.

## Introduction

The ability of specific areas of the brain to process and incorporate information is often intimately dependent upon the body’s hormonal milieu. This milieu can be affected by immediate factors in an organism’s environment (e.g., social interactions, food, weather, population density, time of day), and over a longer temporal scale (e.g., development, season, reproductive period). Focusing on this longer temporal scale, changes in hormones during critical stages in juvenile development can affect cognition, such as that involved with the learning of language/birdsong (Marler et al., 1988; Fusani & Gahr, 2006). However, much less is known about how endocrine changes experienced on a longer temporal scale in adulthood can affect such factors.

Growing evidence suggests an effect of breeding context and associated reproductive hormones on adult learning and behavior. For example, Maney, Cho & Goode (2006) reported that non-breeding female white-throated sparrows (*Zonotrichia albicollis*) implanted with the steroid hormone estradiol (E2) have increased immediate early gene (IEG) response in an auditory forebrain region when hearing conspecific song as opposed to frequency-matched synthetic tones. Controls, however, do not differ in their response to either stimulus. These data suggest IEG induction in the auditory system is selective for song only when plasma E2 exceeds non-breeding levels. In another example, male song sparrows (*Melospiza melodia*) experience rapid effects on intracellular signaling in the brain in response to an acute injection of E2, and these rapid effects differ across the seasons (Heimovics et al., 2012). These and other reports suggest a plastic nature of adult auditory selectivity in response to E2.

To add to our understanding of this emerging paradigm, we examined how differences in seasonality and circulating E2 manifest behaviorally in the adult male songbird, the European starling (*Sturnus vulgaris*). European starlings are open-ended learners, meaning their song repertoire size can increase with age as opposed to crystallizing during development (Eens, Pinxten & Verheyen, 1992). Additionally, they are photoperiodic, seasonal-breeders, breeding in the spring when weather is clement and food is relatively plentiful compared to the winter. Early in the spring, birds become what is termed “photostimulated”, in that they respond to increasing daylight and become reproductively active. Late into the summer, birds become “photorefractory”, in that they stop responding to long days in preparation for winter, ceasing reproductive activity. After experiencing this state of photorefractoriness, birds become “photosensitive” during the winter. In this state, birds are still not reproductively active but are ready to respond to the anticipated increase in light after the winter solstice (reviewed in McGuire, Calisi & Bentley, 2010).

The learning of song can occur not only during the breeding season, but throughout the year (Bohner et al., 1990). This species demonstrates seasonal differences in immediate early gene response to song. Gene expression in an area of the brain involved with song and social behavior (the caudal part of the ventrolateral zone of the lateral septum - LSc. vl) is positively related to song in a breeding context but negatively related to song in a nonbreeding context (Heimovics & Riters, 2005; Heimovics & Riters, 2006; Heimovics & Riters, 2007). Thus, we hypothesized that starlings will exhibit differential behavioral responses to song depending on whether birds were photosensitive, photostimulated or photorefractory, and this difference will be affected by circulating E2 concentration.

Using an operant conditioning two-alternative choice procedure similar to that used previously with this species (Knudsen & Gentner, 2013), we measured the response of birds in different photoperiodic states to conspecific song and manipulated circulating E2 concentrations within each. Specifically, we measured the latency of birds to learn and respond to new song patterns while varying E2 availability during a time of (1) photostimulation, (2) photorefractoriness, and (3) photosensitivity.

We trained birds to discriminate and classify small conspecific song segments (“motifs”). Then, we manipulated circulating estradiol with subcutaneous implants of (1) 17-β E2, (2) fadrozole (FAD), a highly specific aromatase inhibitor that suppresses circulating estradiol concentrations (Wade & Arnold, 1994; Schlinger & Brenowitz, 2002; Remage-Healey, Maidment & Schlinger, 2008), (3) FAD + E2, with the intent of recovering the negative effects of FAD on circulating E2 concentrations, and (4) saline controls. We administered each treatment in random order and measured the (1) total number of motif sets learned, (2) recognition accuracy, (3) acquisition rate of learned motifs, and (4) motivation to complete the task.

## Methods

### Groups

All protocols used in this experiment were approved by and in compliance with the University of California Office of Lab Animal Care and Use (No. S05383). European starlings were wild caught in southern California as juveniles late in the summer and housed in outdoor aviaries with food and water available *ad libitum* until the experiment began. Eleven adult, 1-year-old males served as subjects, and each bird was randomly assigned to one of three groups: (1) photostimulated (*n* = 4), (2) photorefractory (*n* = 4), (3) photosensitive (*n* = 3). Each individual within each group randomly experienced all treatments in a repeated measures design, permitting both within and between subjects comparisons.

### Photoperiod manipulation

The reproductive system of the European starling is directly controlled by photoperiod, and we manipulated light:dark (L:D) schedules in the laboratory, as has been routinely done with this species, to induce the appropriate photoperiodic stage (Stevenson et al., 2009; Stevenson, Bernard & Ball, 2009). In brief, the photosensitive and photostimulated groups were brought into the lab in the winter (post-molt; 10L:14D), when birds were naturally experiencing short days and had entered into a photosensitive state. The photosensitive group was maintained on short days (10L:14D) during training (3 weeks) immediately followed by testing (6 weeks). The photostimulated group was maintained on short days during training (3 weeks) and then immediately transferred to long days (16L:8D) for testing (6 weeks). The photorefractory group was brought into the lab at the end of May, when birds were naturally experiencing long days (14L:10D), and held on long days during training (3 weeks), immediately followed by testing (6 weeks), to maintain a photorefractory state (16L:8D).

To confirm the appropriate photo-inducible state was achieved, we examined circulating testosterone concentration and beak coloration. Both of these factors change in accordance with reproductive state, with bright yellow being indicative of high circulating androgens during the photostimulated period, and greyish-black indicative of low circulating androgens during photorefractory and photosensitive periods. Measuring the change in beak coloration has been shown to be a reliable method for determining general concentrations of circulating testosterone (Gwinner, 1975; Ball & Wingfield, 1987; Falk & Gwinner, 1988; Bernard & Ball, 1995), especially in captive bird populations (Wingfield & Farner, 1978; Millar & King, 1984; Wingfield & Moore, 1987). Immediately prior to and on the last day of testing, we scored beak color on a scale of one to three, with one being dark grey-black and three being bright yellow, and examined whether a significant difference existed in coloration as well as in testosterone concentration between photoperiodic states. We predicted that testosterone would be higher and beak color would be more yellow in photostimulated birds as opposed to photosensitive and photorefractory.

### Behavioral training

We designed a behavioral training procedure similar to one previously used with this species in the same laboratory (Knudsen & Gentner, 2013) in which birds learned to successively discriminate and classify pairs of short, conspecific song segments (“motifs”). We then subjected birds in each group (photorefractory, photosensitive and photostimulated) to a random series of treatments (E2, FAD, FAD + E2, saline control) and examined the effects of each treatment on their ability to learn and behaviorally respond appropriately to motifs.

Birds were acclimatized to individual housing in identical sound-attenuating chambers (Acoustic Systems, ETS-Lindgren) for two days, given food and water *ad-libitum*, and maintained on their appropriate light schedule. On the third day, training began (described in the following paragraph). Birds were naive to all experimental procedures and stimuli. Food was increasingly restricted as the bird trained until its only source of food was obtained by correct responses to auditory stimuli. Water was always available *ad libitum*.

Vocal recordings made previously of 11 adult male European starlings were used to create multiple song stimuli. Each stimulus consisted of approximately 1 s of a unique motif from a single male. The motif library included all motif categories, but we did not control for the relative prevalence of motif categories. It may be that differences across categories are more salient than differences within categories, and future studies may find it beneficial to explore this dimension. However, we do not think this is a confound in the current study; all motif comparisons were chosen at random, and the probability of being presented with a within-category or across-category comparison is equivalent for all experimental groups. All birds were trained on the same initial set of six training stimuli using a custom-built apparatus. After this initial training, novel stimuli were chosen randomly from a library of 295 unique motifs for testing. All birds were randomly exposed to the same set of stimuli.

**Figure 1:**
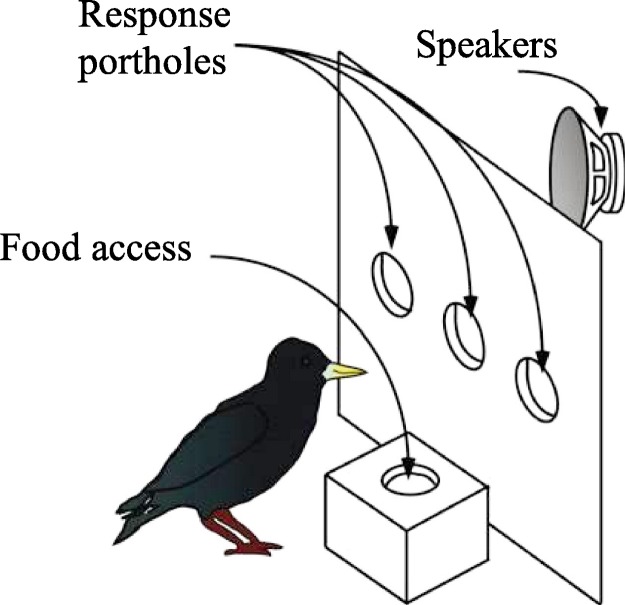
Schematic of acoustic chambers. Birds conducted a two alternative choice (2AC) procedure, initiating a trial by pecking the center porthole. The speaker mounted behind the porthole responded with a particular song motif sound stimuli. Subsequent pecking of the correct left or right porthole in response to the stimuli yielded food from the dish below the porthole panel. An incorrect response was “punished” with no food, and the house light was extinguished automatically for ten seconds. Custom software monitored behavioral responses.

Each bird was trained with a two-alternative choice procedure in which it initiated trials by pecking the middle of three portholes on a wall-mounted operant panel in its chamber (Fig. 1). In each porthole was an infrared transmitter and receiver that detected when the bird broke the plane of the porthole with its beak. This design allowed starlings to probe the hole with their beaks in a manner similar to their natural appetitive foraging behavior (Comins & Gentner, 2010). Pecking the center porthole initiated a trial, at which time one of four song stimuli was played from the speaker, prompting a behavioral response. Two of the song stimuli were taken from a group of six used to initially train the bird. These were presented in order to monitor that the bird was motivated and on task. In addition, two novel stimuli were presented. Two of the four stimuli presented (one previously learned and one novel) required the bird to peck a left of center porthole, and the other two (one previously learned and one novel) required the bird to peck the right of center porthole. If the bird pecked correctly, it was rewarded with food, administered by a remote controlled “hopper” positioned behind the panel, which moved the food in and out of the birds’ reach beneath the opening. This was the only way the bird could receive food. If the bird pecked incorrectly, it was “punished” by lights turning off automatically for ten seconds. If the bird did not peck left or right, it received neither food nor lights out (see electronic Supplemental Information to view trials). The novel song stimuli were considered “learned” when the bird correctly answered 15 of the previous 20 trials for each of the four stimuli that made up a training set. Once learned, a new set of four song stimuli was presented in which, once again, two stimuli were taken from the initially learned six stimuli and two stimuli were novel.

**Figure 2:**
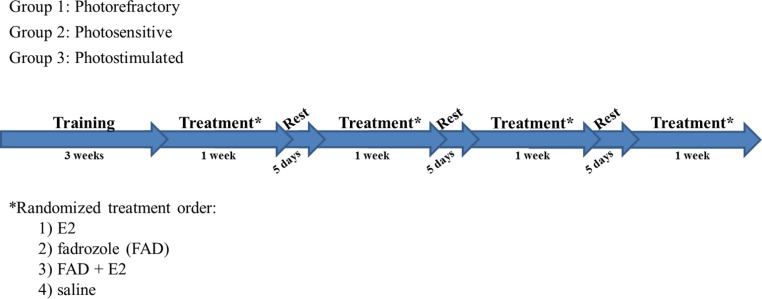
Training and Treatment schedule. Birds were trained for three weeks on the two alternative choice (2AC) procedure. Birds were then given all treatments, one at a time in randomized order (1. E2, 2. Fadrozole (FAD), 3. FAD + E2, 4. Saline) for one week, with five days of no treatment in between.

Birds were trained to this procedure for three weeks before testing began and hormone treatments were administered. After hormonal manipulation, the following day (Day 1) we began measuring the latency for a bird to learn novel sets of stimuli. We continued our observations through Day 7. Implants were removed on Day 8 and no measurements were taken on this day (Fig. 2).

### Song recognition and learning measures

Under each treatment (E2, FAD, FAD + E2, saline control), birds were constantly learning to classify pairs of novel motifs. We measured (1) the total number of motif sets learned during the week of the treatment, and (2) the overall recognition accuracy (mean percent correct), as the total number of times a bird initiated a trial divided by the total number of times the bird chose correctly. We also measured (3) the overall acquisition rate, as the mean number of responses divided by the number of motif-sets required to reach our learning criterion, and (4) the overall response rate, i.e., the number of times a bird initiated a trial but failed to peck after the motif played divided by the total number of trials, which could serve as a proxy for motivation.

### Hormone manipulations

Birds were anesthetized with isoflurane (1%–2%) mixture and subcutaneously implanted with a silastic tube and an osmotic mini-pump over the right or left flank with one of four possible treatments: (1) 17β-estradiol (E2), to increase circulating E2 in the blood, (2) the aromatase inhibitor fadrozole (FAD), to decrease circulating E2, (3) Both E2 and FAD (E2 + FAD) simultaneously, to inhibit the enzymatic conversion of testosterone (T) to E2 and then replace E2, and (4) a saline control. E2 (Steraloids, Newport, RI) was administered in a silastic tube implant (length 12 mm, ID 1.47 mm, OD 1.96 mm, Dow Corning, Midland, MI), and sealed at both ends with silastic glue. Once sealed, implants were soaked for three days prior to implant to avoid a supraphysiological bolus of hormone from being released. FAD, a potent, highly specific inhibitor of aromatase activity known to decrease circulating E2 (Wade & Arnold, 1994; Schlinger & Brenowitz, 2002; Remage-Healey, Maidment & Schlinger, 2008) was administered (8.4 mg/100 µL avian saline) in a micro-osmotic pump (Model 1002; Alzet, Cupertino, CA, USA) with a release rate of 0.25 µl/h (∼21 µg of FAD/h). Solution delivery was verified by comparing pre- and post-implant pump mass. The control treatment consisted of an empty silastic tube implant and a saline-filled micro-osmotic mini-pump. Each bird received all four treatments in random order, with each treatment lasting for one week. The implant was removed on Day 8 and birds were given five days to recuperate before the following treatment was implanted (Fig. 2).

### Hormone assay

We assayed circulating plasma hormone concentrations to confirm effects of artificial photo-manipulation and to validate implant function. We sampled blood from the brachial vein to measure circulating plasma concentrations of (1) T, and (2) E2 via radioimmunoassay (RIA; Wingfield & Farner, 1975; samples were assessed in duplicate).

The first blood sample was taken within one minute of opening sound-proof bird chambers between 11:00 and 12:30 h and was conducted immediately prior to treatment. Blood was centrifuged and plasma was extracted and frozen (at −4°C) until assaying. The second blood sample was collected on the final test day of each treatment to ensure the effectiveness of the implant/treatment.

### Statistical analysis

Data were checked and confirmed for equality of variance and normality, justifying the use of parametric statistics. We performed an ANOVA to examine the relationship between circulating hormone concentrations and photoperiod using JMP, Version 9 (SAS Institute Inc., Cary, NC, 1989–2007). We used nested repeated measures ANOVAs to examine the relationship between photoperiod and performance between and within subjects, with photoperiod as the nested variable. Dependent variables for repeated measures ANOVAs included (1) total number of motif sets learned, (2) recognition accuracy, (3) acquisition rate of learned motifs, and (4) response rate to complete the task. Independent variables were (1) hormone treatment, (2) photoperiod, and the (3) interaction of hormone treatment and photoperiod. Alpha was set at *P* ≤ 0.05. Post hoc tests included paired t-tests for within subjects comparisons, Tukey’s post-hoc test for between subjects comparisons and unpaired t-tests for between group comparisons. Effect size (ES) was calculated by dividing the difference between the means of the two groups being compared by the pooled standard deviation. An ES = 0.20 to 0.49 was considered to indicate a small effect, an ES = 0.50 to 0.79, a moderate effect, and an ES = 0.80 and above, a large effect (Cohen, 1988).

## Results

### Hormones

Recoveries from RIA for T and E2 were 79 and 70%, respectively, with an intra-assay variation of 8.97 and 8.98% and inter-assay variation of 5.26 and 8.48%. The assay detection limit was 0.14 ng/mL for T and 0.12 ng/mL for E2. Raw values are reported in Table 1.

**Table 1:** Mean, raw values according to photoperiod for circulating testosterone (T) and estradiol (E2). Baseline values signify natural circulating concentrations of hormones prior to any manipulation. Control values signify circulating concentrations of hormones while implanted with a control, empty silastic tube and saline-filled osmotic mini-pump.

ng/ml ± SD					
	Baseline	Control	E2	FAD	E2 + FAD
**T**					
Photorefractory	0.08 ± 0.02	0.06 ± 0.02	0.08 ± 0.01	0.08 ± 0.02	0.06 ± 0.01
Photosensitive	0.31 ± 0.18	0.55 ± 0.24	0.10 ± 0.02	1.46 ± 0.75	0.16 ± 0.02
Photostimulated	1.03 ± 0.76	1.33 ± 1.04	0.07 ± 0.01	1.98 ± 1.30	0.08 ± 0.02
**E2**					
Photorefractory	0.13 ± 0.10	0.93 ± 0.88	1.37 ± 0.85	0.10 ± 0.07	0.87 ± 0.59
Photosensitive	0.21 ± 0.11	0.22 ± 0.04	0.92 ± 0.71	0.11 ± 0.01	1.90 ± 1.33
Photostimulated	0.28 ± 0.07	1.72 ± 1.66	1.96 ± 1.73	1.04 ± 0.73	3.07 ± 2.14

**Figure 3:**
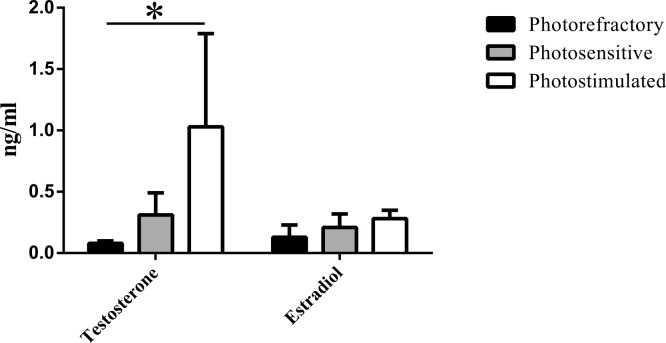
Average baseline (pre-implant) raw values of circulating testosterone (T) and estradiol (E2) concentrations. An asterisk denotes a significant difference.

**Figure 4:**
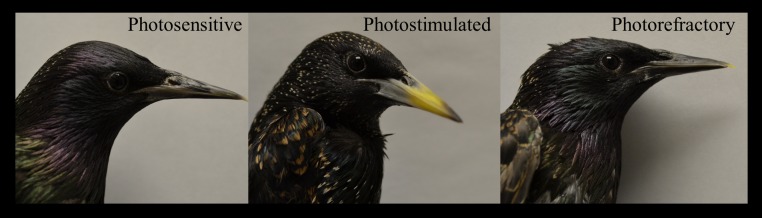
Beak coloration resulting from photo-manipulation. The beaks of the artificially photostimulated birds were more yellow than birds in photosensitive and photorefractory states, indicating successful artificial photostimulation accompanied by an increase in circulating androgens.

Concentrations of baseline circulating T, taken before hormone manipulations, confirm the effectiveness of our photoperiod manipulations (*F* = 4.17, *P* = 0.02; Fig. 3). T was significantly higher in the photostimulated group as compared to the photosensitive and photorefractory groups. Additionally, all photostimulated birds scored a 3 for beak score (yellow) and all photosensitive and photorefractory birds scored a 1 (grey-black; Fig. 4). Circulating E2 did not differ between photoperiods (*F* = 1.67, *P* = 0.23; Fig. 3).

Likewise, our E2 and FAD treatments produced their intended effects. In all groups, there was a significant effect of treatment on circulating E2 (*F* = 4.72, *P* < 0.01), and birds receiving E2 and E2 + FAD treatments had higher E2 concentrations (*P* < 0.01 for each) than birds sampled at baseline. Circulating E2 did not differ between baseline and controls (*P* > 0.05). Circulating concentrations of E2 in FAD-treated birds fell below the detection limit of the assay, meaning concentrations were technically too low to measure. FAD did not increase circulating T compared to baseline or control concentrations (*P* > 0.05 for each).

### Behavioral training

**Figure 5:**
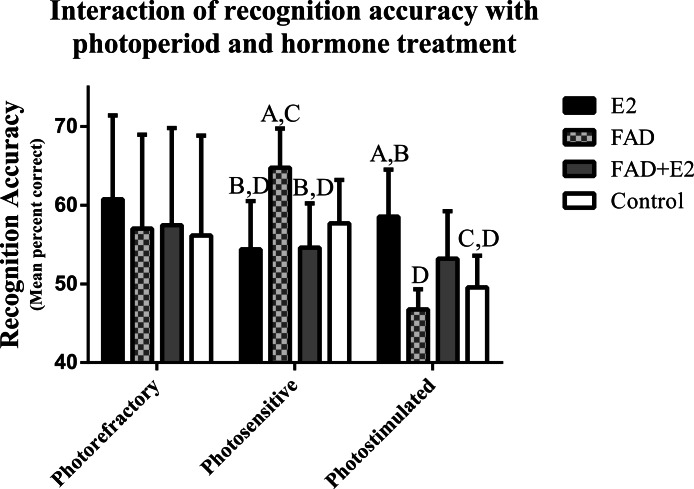
Effects of photoperiod and hormone treatment on recognition accuracy. Standard errors are plotted, and variables labeled with different letters are significantly different. Note: the Y axis begins at 40% to provide a higher viewing resolution of significant differences.

Results from a repeated measures nested ANOVA suggest an interaction effect of recognition accuracy with both photoperiod and hormone treatment (*F*_6,24_ = 2.47, *P* = 0.052). While alpha was originally set at 0.05, we felt a result of *P* = 0.052 warranted further investigation, and we report effect size and results from post-hoc comparisons. Photosensitive birds given FAD had higher recognition accuracy than birds given E2 (*P* = 0.034; ES = 0.92 – large effect) and FAD + E2 (*P* = 0.04; ES = 1.62 – large effect). Conversely, photostimulated birds given FAD had lower recognition accuracy than those given E2 (*P* = 0.01; ES = 1.48 – large effect). Also, E2 treated birds in the photostimulated group had higher recognition accuracy than controls (*P* = 0.03; ES = 0.50 – moderate effect), and those given FAD also had lower recognition accuracy than those given FAD during the photosensitive period (*P* = 0.03; ES = 1.67 – large effect; Fig. 5).

**Figure 6:**
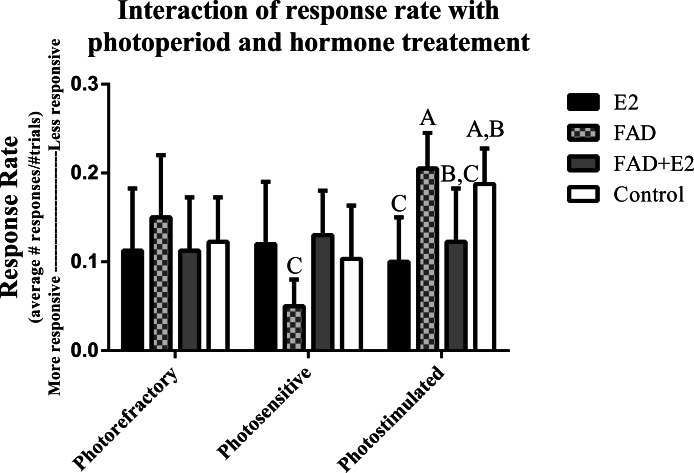
Effects of photoperiod and hormone treatment on response rate. Note: the lower the value, the more responsive an individual was to the task. Standard errors are plotted, and variables labeled with different letters are significantly different.

Results also suggest an interaction effect of response rate with photoperiod and hormone treatment (*F*_6,24_ = 2.447, *P* = 0.054). Again, because of the resulting proximity to alpha, we pursued effect size and post-hoc analyses. Photosensitive birds given FAD had lower response rates than birds given FAD in the photostimulated group (*P* = 0.02; ES = 0.58 – moderate effect size). Photostimulated birds given E2 had a lower response rate than those given FAD (*P* = 0.01; ES = 1.12 – large effect) and controls (*P* = 0.02; ES = 0.64 – moderate effect). Photostimulated birds given FAD + E2 also had a lower response rate than those given FAD (*P* = 0.03; ES = 0.87 – moderate effect) (Fig. 6).

The total number of motif sets learned and acquisition rate were not affected by/interact with photoperiod or treatment (*F*_6,24_ = 0.43, *P* = 0.86; *F*_6,24_ = 0.68, *P* = 0.67, respectively). Baseline E2 did not correlate with any of the learning variables measured (*P* > 0.05 for all), and the order of treatment did not affect any of the learning measures examined (*F*_7,43_ = 0.57, *P* = 0.78).

## Discussion

The results of this study demonstrate how E2 and reproductive state can interact to shape auditory learning in an adult, male songbird, driving recognition in opposite directions in different states. We hypothesized that European starlings would exhibit differential behavioral responses to song depending on the photoperiod, and this difference would be affected by circulating E2 concentration. We found that starlings did not exhibit different responses depending on photoperiod alone, but manipulation of E2 altered responses differently according to the photoperiodic state of the bird. During photostimulation, elevated E2 improved song recognition accuracy and response rates as compared to controls and to birds treated with FAD. Conversely, when birds were photosensitive, reduced E2, resulting from treatment with FAD, was related to improved recognition accuracy and higher responses rates. Due to the time and cost of conducting such an experiment, our sample sizes were small. However, after using a repeated measures design and considering effect size, the significant relationships we see emerging are noteworthy. We will focus our discussion on these results with the intent of inspiring further study to better understand such relationships. Less focus will be spent discussing non-significant results, as small sample size may occlude more subtle relationships from emerging.

We examined four variables to better understand the effects of E2 on auditory recognition and learning: (1) the total number of motif sets learned, (2) recognition accuracy, (3) acquisition rate of learned motifs, and (4) the overall response rate. Recognition accuracy and the overall response rate were the only variables that yielded significant interactions with photoperiod, but many factors may have played a contributing or underlying role in producing such significant interactions. For example, perception, learning capacity, and motivation may have all been affected by E2, resulting in differences in the number of times a bird initiated a trial and carried through with the task (recognition accuracy) or the number of times a bird initiated a trial but failed to peck after the motif played, divided by the total number of trials (the overall response rate). Thus, each variable measured may not have been the direct result of changes in E2, and more consideration for the sources of interaction should be explored in future studies. However, changes in recognition accuracy and response rate under E2 manipulation indicate that ultimately E2 does play a role in auditory recognition and learning.

FAD is a powerful tool commonly used to manipulate and decrease circulating E2 concentrations in the blood. However, being a highly specific aromatase inhibitor, it may inadvertently cause a buildup of androgen precursors resulting from the inhibition of the aromatase enzyme. Because of this, we measured the effects of FAD on T circulation. FAD treatment did not significantly increase circulating T in the blood compared to baseline and control treatments. Unfortunately, androgen circulation in the brain could not be measured during this experiment. Thus, it is conceivable that some of the effects observed during FAD treatment may be attributed to not only low E2, but to high androgens in the brain. However, treatments not involving FAD in which animals were given E2 alone also resulted in significant behavioral changes. This suggests that E2 does play a role in processing and responding to song at this important time of reproductive activity.

Little is known about the effects of circulating estradiol concentrations in adult male starlings as compared to females, but males do circulate E2 throughout the year (Dawson, 1983). However, Dawson (1983) focused his studies of estrogen circulation only in females, and the extent of E2 circulation in wild male starlings, to our knowledge, has yet to be reported. While inter-assay comparisons can prove troublesome, Dawson (1983) reported female starlings to have a circulating E2 concentration of about 1.25 ng/mL when photostimulated. Our males averaged 0.28 ng/mL baseline when photostimulated, and 1.96 ng/mL when treated with E2 at this time. Thus, it is important to note that E2 treatment could have exceeded the physiological range for males. Estrogens do circulate at appreciable levels in both young and adult male songbirds (Adkins-Regan et al., 1990; Weichel et al., 1986; Marler, Peters & Wingfield, 1987; Marler et al., 1988; Harding, Sheridan & Walters, 1983). Changes occur in areas of the brain necessary for song processing and production, such as changes in nuclei volume, cell number, and cell size, over the reproductive cycle (Nottebohm, 2005) and these changes can be sensitive to fluctuations in E2 (Soma et al., 2003; Maney, Cho & Goode, 2006; Remage-Healey et al., 2010; Heimovics et al., 2012).

European starlings have the ability to learn song throughout the year (Bohner et al., 1990). Thus, in the broader context, the learning effects we observed in response to E2 (or lack thereof) could be related to seasonal changes in selective advantages for song-based behaviors that are tied to the types of vocalizations specific to the season. During the breeding season, vocal recognition would predominate (e.g., competition in mate attraction and mate guarding) while, during the non-reproductive period, vocalizations would be more important for social cohesion. Specific to this species, vocalizations appear to depend on the context and the receiver, serving, for example, as an attractant (mate attraction: Gentner & Hulse, 2000; male assessment of potential competitors: Mountjoy & Lemon, 1991) or a repellant (territory defense: Eens, Pinxten & Verheyen, 1993), and these functions likely vary as the breeding cycle unfolds (Eens, Pinxten & Verheyen, 1994). Likewise, the songbird neuroendocrine phenotype is dynamic, with changes occurring in areas of the brain necessary for song processing and production over the reproductive cycle (Nottebohm, 2005). Circulating E2 in the blood and receptors for E2 in areas of the songbird brain involved in song processing and production can increase during the breeding season (Fusani et al., 2000), a time when song is very important for mate attraction and territory maintenance. Thus, higher circulation of E2 at this important time of reproductive activity may yield advantages in processing and responding to song.

Interestingly, baseline, un-manipulated E2 concentrations were not related to auditory recognition and learning measures. This could be for many reasons. For one, such artificial captive housing conditions can negatively affect the circulation of sex steroids, like E2, in the body (Calisi & Bentley, 2009), and more dramatic changes in baseline E2 might be observed when examined in the animal’s natural environment. Future inquiry of this nature may be better served by utilizing a more natural setting as part of the experimental arena. Another possibility is that there may not be a relationship between baseline circulating E2 in the blood and learning. The avian brain can produce its own E2 *de novo*, and mounting evidence suggests that neural E2 can serve as a neuromodulator and/or neurotransmitter, rapidly regulating aspects of auditory coding and discrimination (Balthazart & Ball, 2006; Jeong et al., 2011; Tremere & Pinaud, 2011; Remage-Healey & Joshi, 2012; Tremere et al., 2012). Our manipulations of circulating E2 most likely affected circulation in the brain (Balthazart et al., 2004; Yoder, Lu & Vicario, 2012), though we were unable to measure this directly. Under this logic, this is potentially why we observed changes in auditory recognition, learning and behavior under E2 manipulation rather than in relationship to baseline circulating E2.

Estradiol can affect auditory processing in the songbird brain. It can induce changes in brainstem catecholaminergic cell groups and projections to the auditory forebrain of adult female songbirds (LeBlanc et al., 2007). Riters, Olesen & Auger (2007) report evidence that female endocrine state can influence catecholamine responses, and this interaction is related to female responses to courtship. In female songbirds, immediate early gene responses in the auditory pathway are selective for conspecific male song only when experiencing breeding-typical E2 concentrations, as opposed to non-breeding-typical concentrations (Maney & Pinaud, 2011). Previous work in male zebra finches has shown that inhibition of E2 production in an area of the adult songbird brain involved in auditory processing disrupts the bird’s ability to behaviorally respond to recordings of its own song (Remage-Healey et al., 2010). Additionally, depletion of E2 in this species negatively affects memory for songs (Yoder, Lu & Vicario, 2012). Recent data reported from a seasonally breeding male song sparrow (*Melospiza melodia*) indicate that E2 can have rapid effects on intracellular signaling in the brain, and these effects differ across the seasons (Heimovics et al., 2012). E2 decreased phosphorylated extracellular-signal-regulated kinases (pERK) in the song nucleus of the caudomedial neostriatum (NCM) during the breeding season only. During the non-breeding season, E2 decreased phosphorylated CREB (pCREB) in the medial preoptic nucleus (POM), an area known to be involved with song production as well as aggression and sexual behavior (Balthazart & Ball, 2006; Patil & Brid, 2010; Pan et al., 2010). Additionally, E2 decreased pERK in the hippocampus, an area also involved in auditory processing, during the breeding season, but increased pERK in this area during the non-breeding season.

In our experiment, we observed in male European starlings an increase in auditory recognition and response rates in photostimulated birds in response to E2. Heimovics et al. (2012) posited that the decrease they observed in phosphorylation during the breeding season in response to E2 may be related to rapid auditory discrimination needed during this time of year. Using another songbird model, our data support this hypothesis by showing that an increase in E2 during photostimulation is related to increased auditory recognition and motivation, and that this changes according to photoperiod.

In sum, our data offer more insight and implications for how estradiol and photoperiod may mediate auditory processing in vertebrates. We report that E2 and photoperiod can interact to shape auditory recognition and learning in adults. Understanding how E2 affects auditory recognition and learning during different periods of reproductive and non-reproductive activity in the European starling increases our understanding of auditory processing in the avian brain. It also presents a model to study how endocrine changes could modulate auditory processing in animals in general. Due to the complex nature of auditory learning, the more we know about its interaction with the ever-dynamic endocrine system, the more we understand the mechanisms driving cognitive abilities in general.

## Supplemental Information

10.7717/peerj.150/supp-1 Supplemental Information 1 Behavioral Training Procedure

10.7717/peerj.150/supp-2 Supplemental Information 2 Supplementary Video: Behavioral Training Procedure In this video, a male European starling initiates a trial by pecking the center port. Motif A is heard, and the bird correctly pecks the left port and is rewarded with food. He initiates a second trial, and motif B is heard. The correct behavioral response would be to peck the right port. However, he incorrectly pecks the left port and is “punished” with light outs for 10 s. Song stimuli were considered “learned” when the bird correctly answered 15 of the previous twenty trials for each of the four stimuli that made up a training set.
